# *Sinolatindia petila* gen. n. and sp. n. from China (Blattodea, Corydiidae, Latindiinae)

**DOI:** 10.3897/zookeys.596.8332

**Published:** 2016-06-07

**Authors:** Lu Qiu, Yanli Che, Zongqing Wang

**Affiliations:** 1 Institute of Entomology, College of Plant Protection, Southwest University, Beibei, Chongqing 400716, P. R. China

**Keywords:** Blattodea, China, cockroaches, Corydiidae, Latindiinae, new genus, new species, Sinolatindia, Sinolatindia petila

## Abstract

*Sinolatindia petila* **gen. n.** and **sp. n.** (Blattodea: Corydiidae: Latindiinae) is reported from Yunnan Province, China. Description, illustrations and a distribution map of the new taxon are provided. Comparisons with the type genus *Latindia* Stål, 1860 and the genus *Homopteroidea* Shelford, 1906 are given.

## Introduction

Latindiinae, a group of small sized Corydiidae cockroaches, is a poorly studied subfamily that superficially differs from the typical Corydiidae. Despite superficial differences, the diagnostic character of Corydiidae, “anal area of hind wing usually flat in resting position and simply folded over the anterior field” (Roth 2003), still applies to Latindiinae. Previously, Latindiinae species were generally treated under the subfamily Corydiinae/Corydinae (Kirby 1904; Shelford 1912; Hebard 1917, 1921; Karny 1921; Rehn and Hebard 1927; Hanitsch 1931; Rehn 1932, 1937; Bruijining 1959) or the family Corydidae (Brunner 1865). Then Handlirsch (1925) erected the subfamily Latindiinae to include these small, delicate cockroaches with *Latindia* Stål, 1860 designated as the type genus. This subfamily status was accepted by some researchers (Princis 1950; Rehn 1951), but it was later raised to family level (Brues and Melander 1932; Princis 1960, 1963). Princis (1963) listed twelve genera under the family Latindiidae. Roth (2003) kept the subfamily Latindiinae, but didn’t list the genera under it. Pellens and Grandcolas (2008) followed the subfamily status and listed four Brazilian genera in Latindiinae. Beccaloni (2014) only lists the two genera *Latindia* and *Buboblatta* in the subfamily Latindiinae, and many of the genera listed in Princis (1963) are treated as undetermined genera. Several phylogenetic works (Djernæs et al. 2015; Legendre et al. 2015) have shown Latindiinae as being closely related to Nocticolidae; but due to the limited taxon sampling of Nocticolidae, Latindiinae and other Corydiidae (Djernæs et al. 2015), the current taxonomy is maintained.

Recently, the cockroach collection of the Institute of Zoology, Chinese Academy of Science, Beijing (IZCAS) was examined, and two peculiar cockroach specimens were found. They are very small and delicate. After careful study of these two specimens, it was established that they should be classified as a new species belonging to a new genus under the subfamily Latindiinae. This new genus resembles the type genus *Latindia* Stål and may be confused with the southeast Asian genus *Homopteroidea* Shelford. A comparison is made of this new genus with *Latindia* and *Homopteroidea*.

## Materials and methods

Specimens studied and examined during this research are deposited in the following institutions:

IZCAS
Institute of Zoology, Chinese Academy of Sciences, Beijing, China 


NRM
Swedish Museum of Natural History (Naturhistoriska riksmuseet), Stockholm, Sweden 


OUM
Oxford University Museum of Natural History, Oxford, UK 


Morphological terminology used in this paper mainly follows Hanitsch (1929), Roth (1995a, 1995b) and Klass (1997), and venation terms mainly follow Kukalová-Peck and Lawrence (2004) with the modification by Li and Wang (2015). Original and important taxonomic references are cited after taxon names. Some figures in this article are without scales because the original figures lack scales or are illustrated with magnification.

The venation terms and their abbreviations in parentheses in this article are listed as below: subcosta (Sc), radius (R), radius anterior (RA), presutural vein, media (M), cubitus anterior (CuA), cubitus posterior (CuP), CuP in claval furrow (CuP in cfr), anal fold (afd), anal anterior (AA), and anal posterior (AP). The presutural vein is an important character in *Homopteroidea*, which may be a separated part of CuA, the area between it and the sutural margin totally hyaline, which is known as presutural zone, the terms “presutural vein” and “presutural zone” follow Hanitsch (1929) and Roth (1995a, 1995b).

The current Latindiinae only contains two genera (Beccaloni 2014), and many of the former Latindiinae genera are treated as subfamily undetermined (Roth 2003). It is inadequate to establish this new genus by only comparing with the current two genera, we have carefully reviewed the ten genera of Latindiidae listed in Princis (1963) (excepting *Biolleya* and *Stenoblatta*, which have been moved into Blaberidae by Roth (2003)) and we found *Sinolatindia* gen. n. is very similar to *Latindia*. We also compared the new genus with *Homopteroidea*, since they are all distributed in Oriental Region and they may be confused by some common characters.

The genital segments of the examined specimens were macerated in 10% NaOH and observed in glycerin jelly using a Motic® K400 stereomicroscope and a Leica® M205A stereomicroscope. All drawings were made with the aid of Adobe Photoshop® CS5, a Leica® M205A stereomicroscope and a Motic® K400 stereomicroscope. Photographs of the specimens were taken using a Canon® 50D plus a Canon® EF 100mm f/2.8L IS USM Macro lens combined with Helicon Focus® software; photos of other characters were taken using a Leica® M205A stereomicroscope. All photographs mentioned above were modified in Adobe Photoshop® CS5. The map was downloaded from www.d-maps.com and modified using Adobe Photoshop® CS5.

## Taxonomy

### Subfamily Latindiinae

Taxon classificationAnimaliaBlattodeaCorydiidae

Handlirsch, 1925

Latindiinae Handlirsch, 1925: 491, designated subfamily with one male Latindia sp., mentioning Latindia and Paralatindia as examples; Rehn 1951: 29; Roth and Slifer 1973: 23; Roth 2003: 34, cited as “Latindiinae Beier”; Pellens and Grandcolas 2008: 18; Djernæs et al. 2015: 297.
Latindiidae Brues & Melander, 1932: 81, key to order Blattariae; Princis 1960: 437; Princis 1963: 98, catalogue.
Corydiinae Hebard, 1917: 205; Karny 1921: 191; Rehn and Hebard 1927: 280; Bruijining 1959: 18.

#### Type genus.

*Latindia* Stål, 1860

#### Remarks.

Based on former studies (Handlirsch 1925; Brues and Meander 1932; Rehn 1951), the Latindiinae is characterized as follows: body small, delicate, legs sparsely with spines, cerci long, subgenital plate of female valved or seam divided, venation simple or less branched, tegmina with an irregular network of large cells made by the cross veins, wings with venation reduced but not as extreme as in Holocompsinae.

This subfamily is badly in need of revision. First, recent molecular phylogenetic studies (Djernæs et al. 2015; Legendre et al. 2015) suggest that the subfamily may be more closely related to Nocticolidae than other Corydiidae. Second, although Princis (1963) listed twelve genera in Latindiidae (now Latindiinae), the validity of these genera has not been verified. What’s more, Roth (2003) moved the twelve genera listed in Princis (1963) out of Latindiinae, and kept ten of them in Polyphagidae (now Corydiidae) as subfamily undetermined. This management is also unreasonable, which made Latindiinae without any genera. Third, the definition of Latindiinae is too simple, a careful study on the type genus especially the male genitalia must be done to redefine Latindiinae.

### Genus Sinolatindia gen. n.

Taxon classificationAnimaliaBlattodeaCorydiidae

http://zoobank.org/14E2B5FE-322F-42BF-8BA5-E2C253129AB2

Sinolatindia 纤蠊属

#### Type species.

*Sinolatindia petila* sp. n. 素色纤蠊

#### Diagnosis.

**Male.** Small size, form elongate elliptical. Body flat, gracile, pubescent. Head transversely triangular, eyes wide apart, interocular space greater than the distance between antennal sockets, ocelli missing. Pronotum suboval, pubescent. Front femur short and robust, type C_1_ spination (Fig. 4C), tarsal claws symmetrical, serrated. Tegmina and wings fully developed, right tegmen with wide, hyaline zone, CuA of wings with 2-3 branches. Supra-anal plate symmetrical, transverse, cerci long. Subgenital plate simple, styli similar. Genitalia complex, with a very elongate L3.

This genus is very close to the type genus *Latindia* Stål, 1860. We have examined the type specimen of *Latindia maurella* Stål, 1860 (Fig. 2G–I. Deposited in NRM, the type species of *Latindia*) and one *Latindia dohrniana* Saussure & Zehntner, 1894 (Fig. 2C–D. Deposited in NRM, determined by Rehn in 1930). Along with the descriptions (Rehn and Hebard 1927; Rehn 1937, 1951), it was found that *Sinolatindia* can be distinguished from *Latindia* by the following characters: 1) pronotum subtransparent, disc without a Y-shaped sulcation (Fig. 2A), whilst in *Latindia*, pronotum rough, median with a distinct Y-shaped sulcation (Fig. 2C); 2) right tegmen with a hyaline area (Fig. 4G), while not with a hyaline area in *Latindia* (Rehn & Hebard, 1927); 3) in tegmina, CuA with transverse branches that generally parallel with CuA (Fig. 4F–G), while branches of CuA are oblique, paralleled to each other in *Latindia* (Fig. 2L). In addition, *Latindia* is restricted to north and south America, while *Sinolatindia* gen. n. is found in East Asia.

This genus may be confused with *Homopteroidea*, both of which have hyaline part of right tegmen and serrated tarsal claws, and all distributed in Oriental Region. *Homopteroidea* used to be determined as a member of Latindiidae (Princis 1963), but Roth (1995a, 2003) treated it as subfamily undetermined. We have examined some *Homopteroidea* collections that were studied by Roth (all deposited in OUM) and in combination with the description (Roth 1995a), we found *Sinolatindia* can be distinguished from *Homopteroidea* by the following characters: 1) head wide, vertex nearly truncated (Figs 2B, 4A), without ocelli (Fig. 4A), while head long, vertex round, with reduced ocelli (Fig. 2F) in *Homopteroidea*; 2) body subtransparent, pubescent, while body horny, smooth and shining, sometimes with a few setae in *Homopteroidea*; 3) venation of tegmina and wings not distinct, right tegmen without presutural vein and presutural zone, but with a large transparent part, the boundary between the colored part and transparent part unclear (Fig. 4G), while venation clear with dark coloration, presutural vein present, right tegmen with a hyaline presutural zone (Fig. 2J–K, except in *Homopteroidea aberrans*, see Roth 1995a and 1995b) in *Homopteroidea*; 4) left phallomere with L3 very elongate, apex curved (Fig. 5B–C), while L3 short, apex usually like a sickle (Fig. 5E–F) in *Homopteroidea*.

**Female.** Unknown.

#### Distribution.

China (Yunnan).

#### Etymology.

This generic epithet comes from the Latin word “*Sino*” and “*Latindia*”. “*Sino*” refers to China, “*Latindia*” in reference to the genus being similar to the Latin American genus *Latindia*.

#### Remarks.

This genus contains all the diagnostic characters of Latindiinae. It also quite resembles *Latindia*. Both of the genera hold in common the following characters: 1) body small, form elongate elliptical, very flat, pubescent; 2) head transversely triangle, eyes wide apart, ocelli absent (Fig. 2B, D); 3) pronotum suboval, with hind margin truncated (Fig. 2A, C); 4) femur stout; arolia absent; 5) tegmina with irregular network of large cells made by cross veins, Sc without branches, wings with venation slightly reduced, only the first AP (known as axillary in Rehn 1951) branched. Based on Rehn (1951), this genus should belong to tribe Latindiini whose Sc of tegmen is free from R, and M is stalked basally with CuA, thus this tribe current with two genera, viz. *Latindia* and *Sinolatindia*.

### Sinolatindia petila sp. n.

Taxon classificationAnimaliaBlattodeaCorydiidae

http://zoobank.org/46E73A6D-3BA1-41CF-9C06-974BD02FE49F

**Figure 1.**
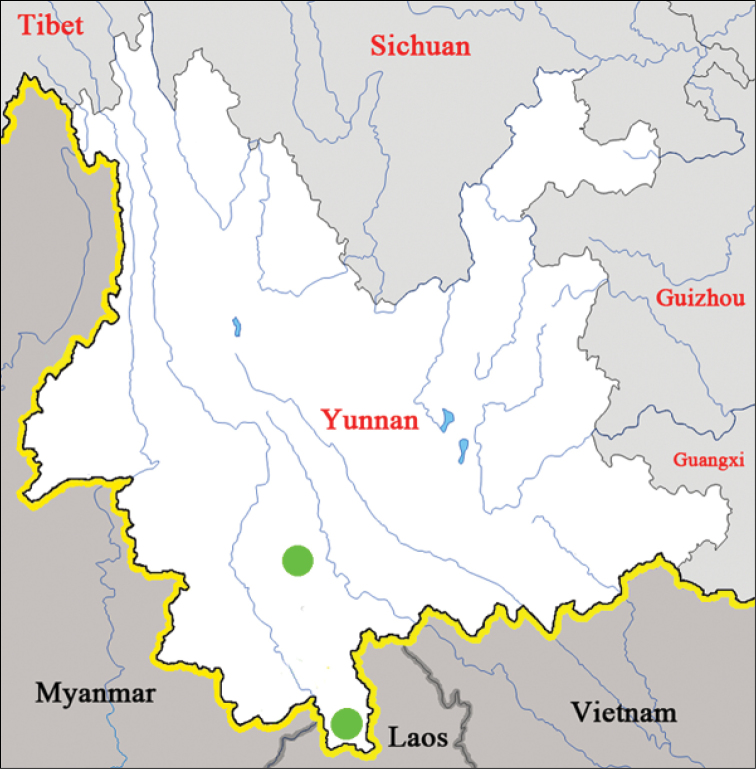
Recorded distribution of *Sinolatindia petila* gen. n. and sp. n.

**Figure 2.**
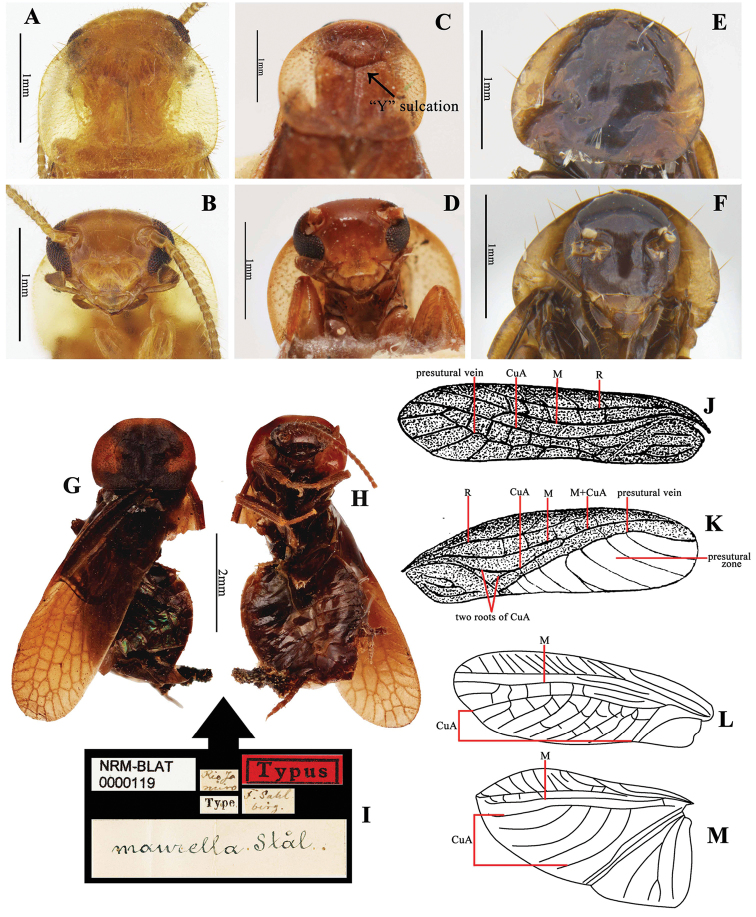
**A–F** Pronotum and head features **A** pronotum of *Sinolatindia petila*, holotype **B** head of *Sinolatindia petila*, holotype **C** pronotum of *Latindia dohrniana* **D** head of *Latindia dohrniana* **E** pronotum of *Homopteroidea minor*, lectotype **F** head of *Homopteroidea minor*, lectotype **G–I** *Latindia maurella*, holotype, female (originally reported as male, but latter corrected as female (Rehn, 1937)) **G** in dorsal view **H** in ventral view **I** label **J–K** original figures of *Homopteroidea shelfordi*, from Hanitsch, 1929 **J** left tegmen **K** right tegmen **L–M** *Latindia dohrniana*, after Rehn 1951 **L** tegmen **M** wing **C–D** and **G–I** photographed by Gunvi Lindberg, Swedish Museum of Natural History, Stockholm (NRM) **E–F** photographed by Katherine Child and provided by Amoret Spooner, Oxford University Museum of Natural History, Oxford (OUM).

**Figure 3.**
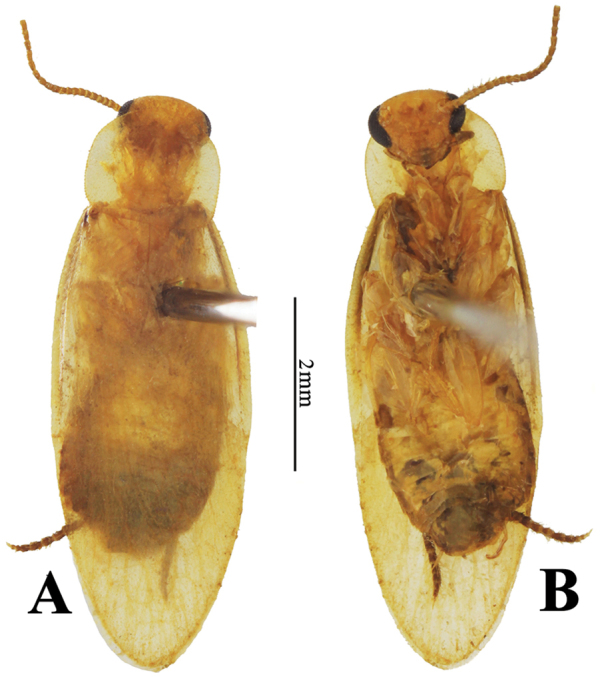
**A–B** *Sinolatindia petila* gen. n. and sp. n., male **A** paratype, in dorsal view **B** same, in ventral view.

**Figure 4.**
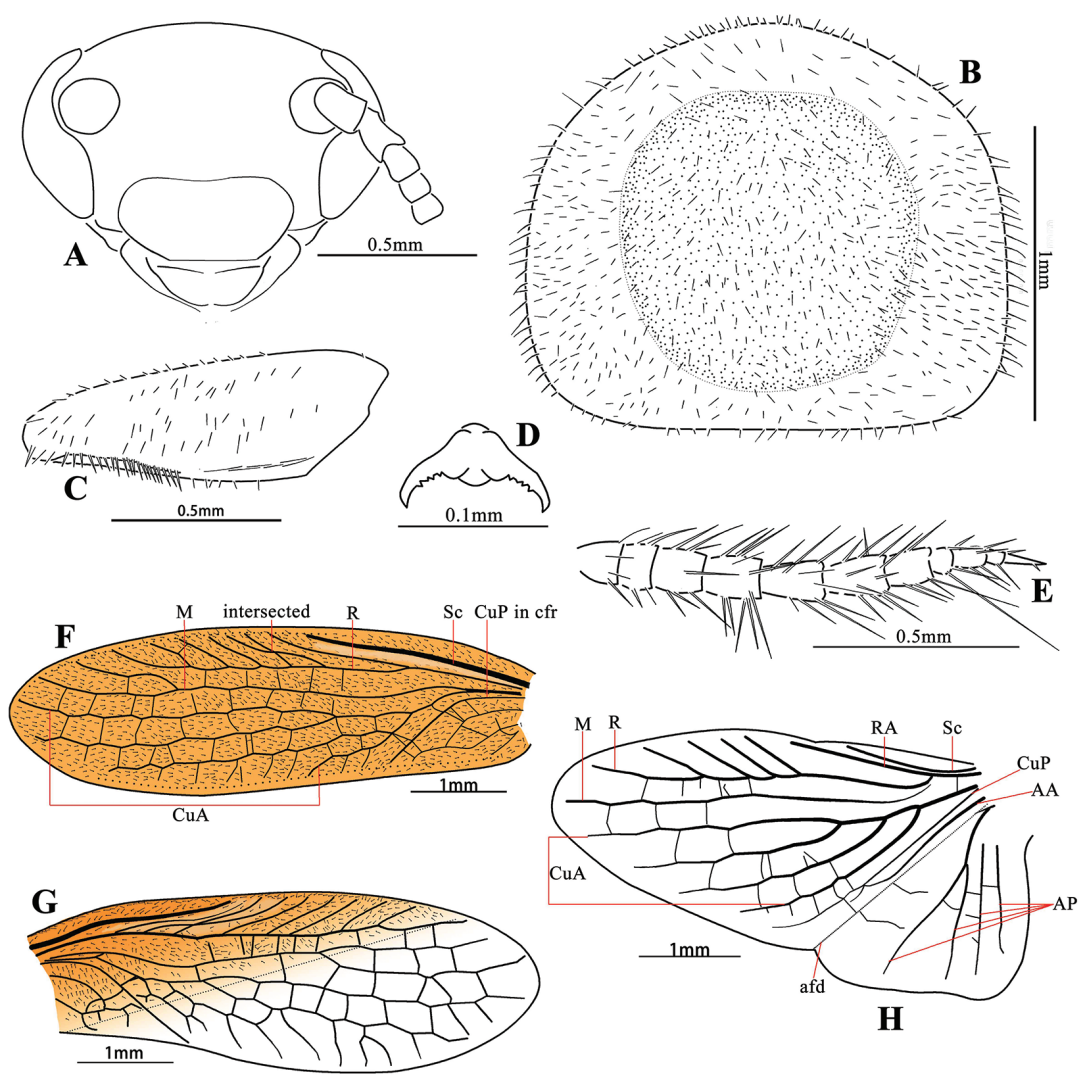
Features of *Sinolatindia petila*, male **A** head **B** pronotum **C** front femur **D** tarsal claw **E** cercus **F** left tegmen **G** right tegmen **H** wing.

**Figure 5.**
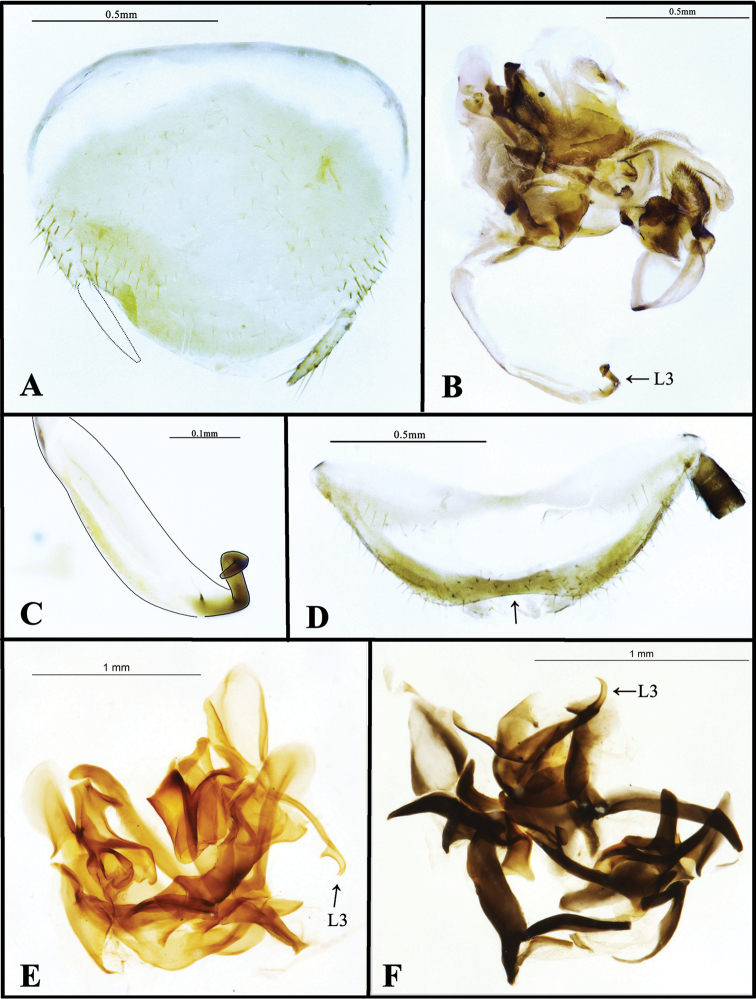
**A–D** Male features of *Sinolatindia petila* **A** Subgenital plate, in ventral view **B** genitalia, in dorsal view **C** L3, in dorsal view **D** supra-anal plate, in dorsal view **E–F** male genitalia of *Homopteroidea* spp. **E** *Homopteroidea nigra*, in dorsal view (this is the original position of the drawing pictured in Roth (1995a), and Roth said it was “dorsal”) **F** *Homopteroidea brachyptera*, holotype, in dorsal view (annotation the same as 5E) **E–F** photographed by Katherine Child and provided by Amoret Spooner, Oxford University Museum of Natural History (OUM).

Figs 1 , 2A-B , 3 , 4 , 5A–D

Sinolatindia petila 素色纤蠊

#### Type material.

**Holotype: Yunnan**: ♂ (IZCAS), 40 km from southeast Jinggu County (景谷县), Puer City, 1000m, 13.V.1957, D. V. Panfilov leg.; **Paratype: Yunnan: **1♂ (IZCAS), Mengla County (勐腊县), Xishuangbanna Prefecture, 620-650m, 2.VI.1959, Suo-Fu Li leg.

#### Diagnosis.

As for the genus (*vide supra*)

#### Description.

**Male.** Body length 5.9–6.0 mm; overall length including tegmen 6.8–7.0 mm; pronotum length × width 1.2–1.3 × 1.5–1.6 mm.

**Coloration**: Body generally light brownish yellow, transparent (Fig. 3A–B). Head yellowish brown, eyes black, antenna brown. Pronotal disk brownish yellow, with hyaline anterior, posterior and lateral areas (Fig. 2A). Left tegmen brownish yellow, right tegmen brownish yellow with wide hyaline area (Fig. 4F–G). Wings hyaline, distal portion light brownish yellow. Venation of tegmina and wings light-colored. Legs brownish yellow. Cerci brown.

Body very flat, narrow, well pubescent. **Head**: exposed dorsally, triangular, longer than its width, vertex nearly straight, face flat, eyes lateral, wide apart, surface with the individual facets convex, interocular space greater than the distance between antennal sockets, ocelli absent (Figs 2B, 4A). **Pronotum**: suboval, pubescent, anterior margin slightly protruded, lateral of anterior margins oblique, lateral margins nearly parallel, hind margin truncated, with lateral corners bluntly rounded (Figs 2A, 4B). **Tegmina and wings**: fully developed extending beyond the end of abdomen, venations not distinct. Tegmina pubescent except the hyaline region of right tegmen, both tegmina with free, long, simple and strong Sc, R with 7-8 oblique branches, the second and third branches intersected (Fig. 4F–G), RA simple, M bifurcated distally, and stalked with CuA basally, CuA with 2-3 branches, major veins reticulate with some cross veins, forming many polygonal cells (Fig. 4F–G). Wings with Sc shorter than RA, M simple, or bifurcate distally, CuA with 2-3 branches, reticulate with very a few cross veins, CuP slender, AA connects CuP medially or distally, the first AP bifurcate (Fig. 4H). **Legs**: Pubescent, front femur stout, apex of the hind margin with one small spine on each side, and followed with contiguous spinules (type C_1_), pulvilli and arolia absent, tarsal claws small, symmetrical, slightly serrated (Fig. 4C–D). **Abdomen**: Supra-anal plate in dorsal view transverse, symmetrical, apex widely depressed (see the arrow in Fig. 5D), anterior and lateral margins pubescent, median hyaline broadly (Fig. 5D), cerci slender, well pubescent, apex acute (Fig. 4E). Subgenital plate generally symmetrical, pubescent, lateral parts with distinctly longer and thicker setae, apex slightly protruding, styli similar, with several long setae (Fig. 5A). **Genitalia**: Very complex, as Figure 5B. Left phallomere with a very elongated L3, the apex of which is curved rectangularly three times as in Figure 5C.

**Female.** Unknown.

#### Distribution.

China: South Yunnan (Fig. 1).

#### Etymology.

The species epithet is from the Latin word “petilus” meaning thin and little in reference to its narrow and small body.

## Supplementary Material

XML Treatment for Latindiinae

XML Treatment for Sinolatindia

XML Treatment for Sinolatindia petila
